# Neuroimaging of Sudden Unexpected Death in Epilepsy (SUDEP): Insights From Structural and Resting-State Functional MRI Studies

**DOI:** 10.3389/fneur.2019.00185

**Published:** 2019-03-05

**Authors:** Luke A. Allen, Ronald M. Harper, Samden Lhatoo, Louis Lemieux, Beate Diehl

**Affiliations:** ^1^Department of Clinical and Experimental Epilepsy, UCL Queen Square Institute of Neurology, London, United Kingdom; ^2^Epilepsy Society MRI Unit, Chalfont St Peter, London, United Kingdom; ^3^The Center for SUDEP Research, National Institute of Neurological Disorders and Stroke, Bethesda, MD, United States; ^4^Department of Neurobiology, David Geffen School of Medicine at UCLA, University of California, Los Angeles, Los Angeles, CA, United States; ^5^Brain Research Institute, University of California, Los Angeles, Los Angeles, CA, United States; ^6^Department of Neurology, University of Texas Health Sciences Center at Houston, Houston, TX, United States

**Keywords:** biomarkers, SUDEP, MRI, functional connectivity, structural imaging biomarkers

## Abstract

The elusive nature of sudden unexpected death in epilepsy (SUDEP) has led to investigations of mechanisms and identification of biomarkers of this fatal scenario that constitutes the leading cause of premature death in epilepsy. In this short review, we compile evidence from structural and functional neuroimaging that demonstrates alterations to brain structures and networks involved in central autonomic and respiratory control in SUDEP and those at elevated risk. These findings suggest that compromised central control of vital regulatory processes may contribute to SUDEP. Both structural changes and dysfunctional interactions indicate potential mechanisms underlying the fatal event; contributions to individual risk prediction will require further study. The nature and sites of functional disruptions suggest potential non-invasive interventions to overcome failing processes.

## Introduction

Sudden unexpected death in epilepsy (SUDEP) is the leading cause of untimely death in epilepsy (1), with a 20-fold increase in incidence over that of sudden death in the general population (2, 3). SUDEP is largely sleep-bound (around 60% of events) and occurs unwitnessed in nearly 90% of cases (4). With no structural, or toxicological indicators of the cause of death, precise underlying SUDEP mechanisms remain elusive.

Observational studies within epilepsy monitoring units (EMUs) show autonomic and respiratory dysfunction preceding SUDEP. A comprehensive assessment of the incidence and mechanisms of cardiorespiratory arrests in EMUs (5) revealed severe alterations to cardiac and respiratory function in the post-ictal period of generalized tonic-clonic seizures (GTCS) which led to SUDEP (*n* = 10 cases). Specifically, transient cessations in breathing preceded terminal apnoea, and ultimately terminal asystole. Prolonged peri-ictal apnea (6), with or without bradycardia and asystole, and post-convulsive central apnea (7) may play a role in SUDEP risk. Cortical and sub-cortical structures that modulate autonomic and breathing processes are of great interest to pre-mortem risk identification through imaging (8), particularly since electrical stimulation studies confirm the role of brain regions often involved in epileptic seizures (9). Sustained post-ictal hypotension is also associated with GTCS (10), further indicating alterations to central autonomic control processes following GTCS. Overall, this key evidence demonstrates centrally-mediated disruption to autonomic and breathing regulatory processes following GTCS (5, 10) and cases of observed SUDEP (5).

GTCS are the leading SUDEP risk-factor (11); experiencing three or more seizures of this type per year is associated with the largest increase in risk (12). The possibility that seizures, especially GTCS, propagate to, and rapidly involve, central autonomic and respiratory brain sites, leading to dysfunction, has been previously hypothesized (13, 14); yet, this central issue to SUDEP remains unresolved.

Neuroimaging is a powerful tool to explore structural and functional brain alterations within distinct sites and networks crucial for autonomic and respiratory regulatory processes in patients who (after being scanned) succumb to SUDEP. Such assessments allow the investigation of structural (tissue) abnormalities or disrupted networks related to SUDEP, and have the potential to shed light on underlying mechanisms and provide biomarkers to prospectively identify living patients at heightened risk. In the following sections, evidence from structural and functional magnetic resonance imaging (MRI) investigations into SUDEP will be discussed, together with potential interventions to overcome deficient processes.

## Evidence From Structural MRI

Structural MRI enables the identification and characterization of brain tissue abnormalities, regional alterations in brain volume, cortical thickness and morphometry, and abnormal structural connections (fiber tracts). Such techniques have been widely applied to epilepsy (15–17), and have the potential to improve understanding of underlying brain physiology and highlight quantifiable disease biomarkers (18, 19). Although the precise pathological mechanisms of SUDEP are not known, some imaging studies have highlighted structural changes to cortical, sub-cortical, and brainstem structures in those who subsequently succumbed to SUDEP and those at greatest risk, indicating morphological disturbances among sites involved in central autonomic and respiratory regulation. In the remainder of this section we provide an overview of the main relevant imaging findings, and interpret them in relation to other, independent work.

### Tissue Loss in Thalamic, Brainstem, and Frontal Sites

Voxel-based morphometry (VBM) has been used to investigate regional gray matter changes in subjects who later died from SUDEP (*n* = 12) and comparable high-risk, low-risk, and healthy controls (20). Gray matter volume of the bilateral posterior thalamus (pulvinar nuclei) was found to be reduced in SUDEP cases and those at high-risk, compared with healthy, and low-risk controls. Although correction for multiple comparisons was not employed in this study, more recent work (21) confirmed posterior thalamic loss (though confined to the left only) in a larger cohort (*n* = 25 SUDEP cases) which employed family-wise-error rate (FWER) correction of *p*-values. Thalamic volume loss in patients who experience GTCS, and therefore who are at greatest risk of SUDEP, has been widely demonstrated (22–24), including loss specifically within the pulvinar (22). We note that in congenital central hypoventilation syndrome, a condition involving breathing and cardiovascular dysfunction, blood flow responses to hypoxia and hypercapnia were found to be altered in the posterior thalamus (25, 26), further supporting its role in mediating control of breathing (27, 28). In other conditions involving impaired autonomic and respiratory function, such as obstructive sleep apnoea (29) and heart failure (30), posterior thalamic volume loss also appears. Posterior thalamic loss raises the possibility that strategic control of low oxygen and CO_2_ is at risk, a serious handicap during ictal events where recovery from low oxygen and high CO_2_ necessitates appropriate responses to such ventilatory conditions.

A recent investigation into neocortical morphometry in patients with GTCS (*n* = 53) revealed widespread thinning, most prominently within the frontal lobe, including orbitofrontal sites, which are involved in cardiovascular regulation (31), and in temporal and parietal cortices (32). The results of volumetric studies are consistent with these findings, revealing tissue loss within the frontal cortex (23), including medial and lateral orbitofrontal regions (22) in patients with GTCS. Those cortical changes should be viewed in the context of volume changes in thalamic sites, since sensory information classically synapses in the thalamus before projecting to cortical sites, with reticular thalamic sites providing an aspect of focus to afferent input. Many of these projections are reciprocal, providing a basis to induce structural alterations in subcortical areas following changes in cortical thickness.

In addition to changes among cortical and sub-cortical structures, more-caudal brain alterations have also been identified in cases of SUDEP. VBM revealed reduced volume of the periaqueductal gray [PAG; (21, 33)]. Volume loss also appears in the medulla oblongata, which becomes progressively more extensive the closer to SUDEP from MRI (34). Portions of the medulla form the final common pathway for cardiovascular and respiratory control. The PAG plays a significant role in cardiorespiratory patterning and recovery; deficient post-ictal PAG-driven compensatory mechanisms have been linked to SUDEP in a mouse model (35). That role stems from projections from forebrain areas, including the amygdala, and its own projections to parabrachial and ventrolateral regions for breathing control (36); concerns of PAG contributions to breathing partially stem from susceptibility of its neurons to opiates (37), with their well-known depression of breathing. PAG neurons show time-locked relationships to both the respiratory (38), and cardiac (39) cycles, as revealed by animal studies, and these relationships are *sleep-state dependent*. In this context, the fact that SUDEP appears preferentially during sleep emphasizes the need to study any functional connectivity changes with the PAG in the context of state change.

Overall, there is accumulating evidence of widespread structural loss, particularly within anatomic regions related to cardiorespiratory functions such as thalamic, frontal lobe (including medial and orbital divisions) as well as brainstem sites in people who suffered SUDEP and in those at greatest risk.

### Tissue Gain in Limbic, Insula, and Sensory Sites

In addition to regional reductions, regional increased volume, and cortical thickness in key autonomic, breathing, and sensory sites have been observed in SUDEP cases and those at high risk. Compared with low-risk and healthy subjects, cases of SUDEP and those at high risk show increased gray matter volume of the right amygdala and anterior hippocampus (20), which are known to be involved in breathing regulation (40). More recent imaging work demonstrates enlargements to additional anterior limbic structures, including the bilateral amygdala, parahippocampal gyrus and entorhinal cortex (21) in SUDEP cases and high-risk subjects. The subcallosal cortex, a region involved in blood pressure regulation (8), is also enlarged—but only in those who subsequently died (21). Bilateral increased mesial temporal structure volumes, including the amygdala, appear in a sub-type of mesial temporal lobe epilepsy (m-TLE) who also had poor post-surgical outcome (41). We note that patients in whom surgery has failed to reduce seizure frequency encompass the group at greatest risk of SUDEP risk, when compared with population-based incidence cohorts, prevalence cohorts, populations from epilepsy clinics, and even refractory epilepsy cohorts (1). Increased volume may reflect gliosis or inflammation, potentially resulting from ongoing hypoxic damage (42) occurring following seizures (43), although this must be confirmed in human epilepsy studies. Uncontrolled GTCS, often seen in subjects who die and those at high-risk, could accelerate such processes, although further work is required to confirm this process.

Patients who experience GTCS also show cortical thickening across a number of sites (32): The post-central gyri, anterior insulae and the subgenual, anterior, posterior, and isthmus cingulate exhibited cortical thickening in GTCS patients (*n* = 53) compared with healthy controls (*n* = 530). While patients who experience GTCS are at highest risk of SUDEP, assessments of cortical thickness are needed in patients who died from SUDEP, since studies including only at-risk populations are complicated by limited interpretability. Elevated volume and cortical thickness are traditionally considered as being linked to improved function or compensatory mechanisms, such as the increased volume within visual cortex observed in deaf vs. hearing individuals (44), and elevated peripheral V1 volume in those with macular degeneration (45). In the context of seizures, however, the explanation for increased volume and thickening is poorly developed, and further investigation is required. For SUDEP, elevated volumes in selected areas, e.g., the amygdala and subcallosal regions, raise concerns; if the increased volumes indeed reflect enhanced function, then the potential for those structures to induce apnea (amygdala) or hypotension (subcallosal region) may place the patient at risk.

### Summary of Structural Imaging Findings

**Figure 1:**
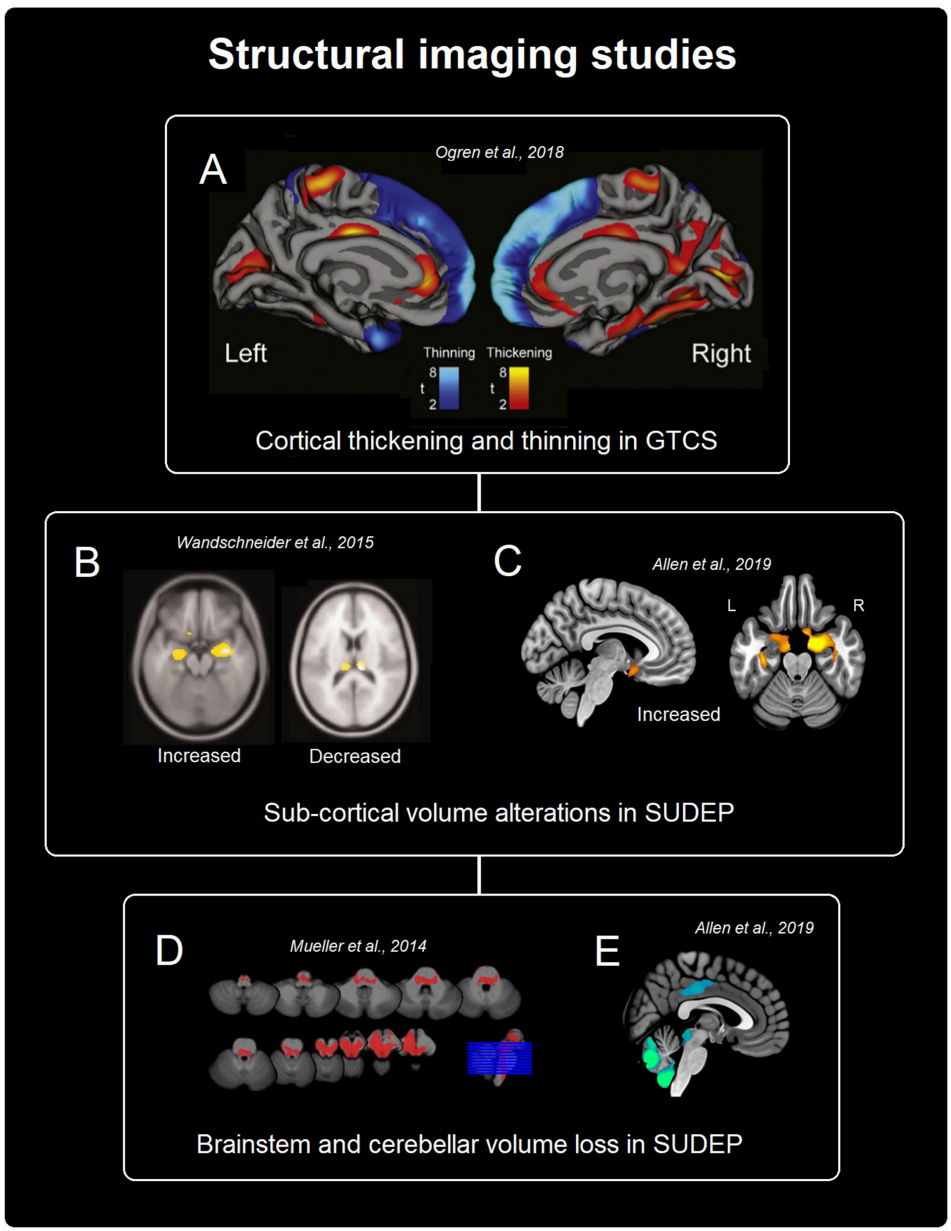
Summary of structural findings from imaging studies in SUDEP and populations at high-risk of SUDEP. **(A)** Shows cortical thickness changes in patients with GTCS (32). **(B,C)** Show sub-cortical gray matter alterations in SUDEP [(20), **B** and (21), **C**]. **(D,E)** Depict brainstem and cerebellar volume loss related to SUDEP [(33), **D** and (21), **E**].

Evidence from morphometry and cortical thickness studies in SUDEP and at-risk groups (i.e., patients with GTCS) demonstrates reduced volume and cortical thinning in thalamic (primarily within posterior portions), frontal (medial and orbital cortex), and midbrain/cerebellar/brainstem sites (Figure 1). Increased volume and regional cortical thickness appear in limbic regions, primarily anterior mesial temporal, especially the amygdala, and cingulate structures, the insula, and sensory areas (Figure 1). Overall, the highlighted volumetric alterations indicate structural injury to key autonomic and respiratory control pathways, including cortical, sub-cortical, and caudal structures; therefore, a possible interpretation is that these abnormalities reflect a mechanism that increases the risk for dysfunction, particularly in circumstances under which autonomic and respiratory processes are challenged, such as during and after GTCS (5). However, a causal link between volume changes and autonomic and respiratory dysfunction is yet to be established in the SUDEP literature and further work is required to elucidate the relationship between volumetric changes and the extent of autonomic and respiratory dysfunction. Further studies which utilize segmentations of regional structures to validate differences in volume are required to overcome the constraints of the typically limited sample size of SUDEP studies.

## Evidence From Resting-State fMRI

Resting-state (RS) fMRI is a brain imaging technique in which subjects undergo fMRI scanning while lying “at rest” in the sense that they are not subjected to any experimental stimulus or task; they are usually asked to lie quietly and stay awake, with their eyes closed. Although it has been argued that the “rest state” in question lacks specificity, this technique has the advantage of being applicable to a wide range of subjects, such as those incapable of performing a specific task [such as in comatose individuals, i.e., (46)] and thus has become an important tool in the study of the patterns of functional connectivity [FC; (47)]. FC describes the connectivity between spatially distant neurophysiological events which share functional properties (48, 49). FC is based on the temporal correlations of spontaneous (i.e., resting state) BOLD (blood oxygen level dependent) fMRI signal fluctuations between regions. From these measures, resting brain functional connectivity can be explored in multiple ways, the extent of which will not be covered in this short article [for a comprehensive review, see (50)]. In the following we review the main findings of this type of study in relation to SUDEP.

### Altered Connectivity Between Central Autonomic and Respiratory Sites

**Figure 2:**
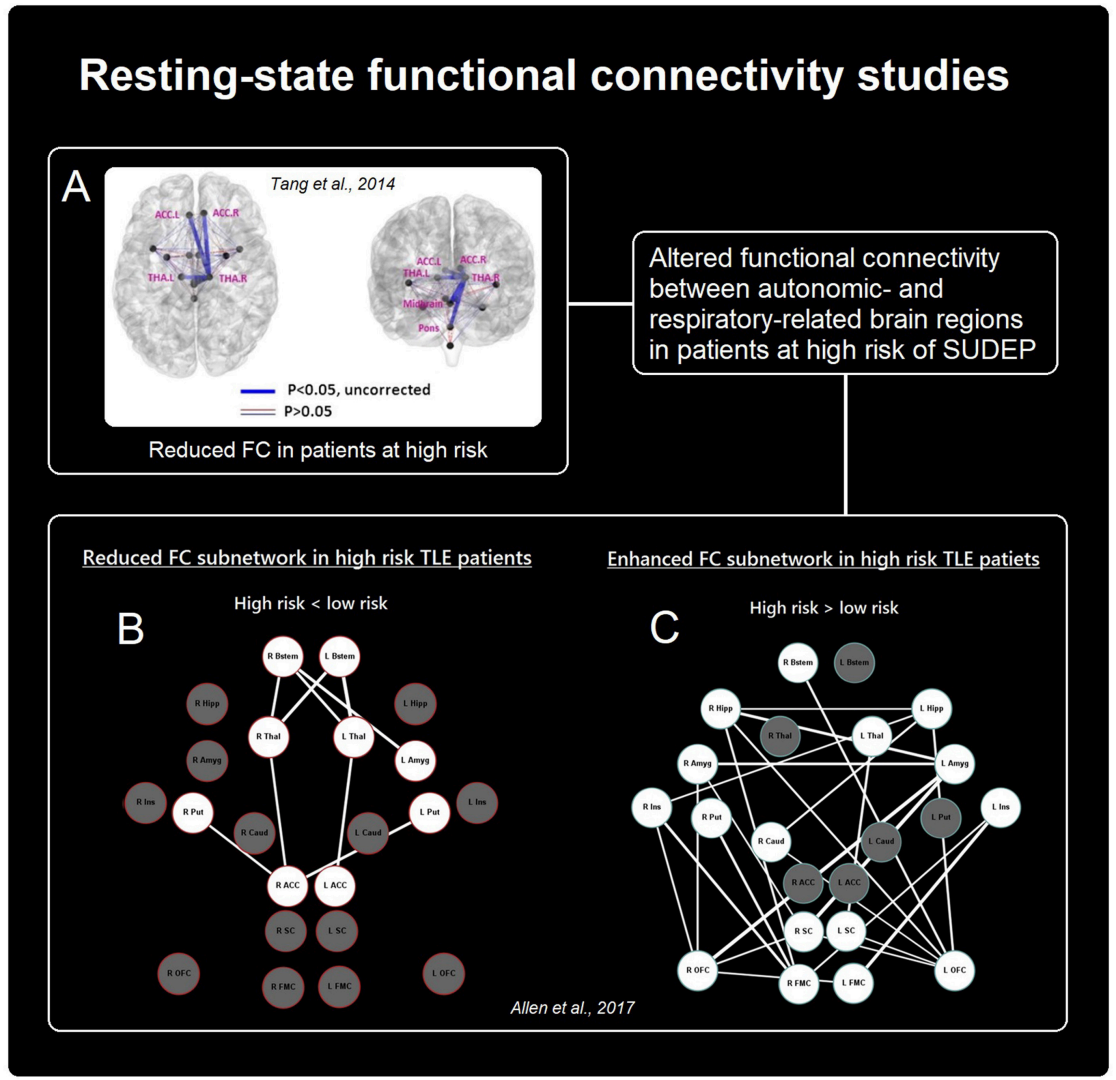
Summary of rs-FC findings in patients at risk of SUDEP. Altered connectivity between cortical and sub-cortical autonomic- and breathing-related sites. **(A,B)** Show reduced functional connectivity in patients at high risk [Adapted from (51), **A** and (52), **B**], while **(C)** Shows increased connectivity between primarily frontal and limbic sites in those at high-risk [Adapted from (52)].

To date, two studies using RS-fMRI have focused on the FC between brain regions related to central mediation of autonomic and respiratory processes in patients with epilepsy (a summary of results is illustrated in Figure 2). Tang et al. (51) compared FC between 13 brain structures (medulla, midbrain, pons, and bilateral amygdala, hypothalamus, thalamus, insula, and anterior cingulate) in relation to SUDEP risk in *n* = 25 patients. High-risk patients exhibited reduced FC between the pons and right thalamus, midbrain and right thalamus, bilateral anterior cingulate and right thalamus, and between the left and right thalamus. In another study Allen et al. (52) in *n* = 32 patients with TLE demonstrated reduced FC between the brainstem and thalamus, and thalamus and anterior cingulate, as well as reductions between the right anterior cingulate and bilateral putamen in high-risk subjects, relative to low-risk subjects. In addition, elevated FC was shown, primarily involving connections between the bilateral medial/orbital frontal cortices and bilateral mesial temporal structures (hippocampus and amygdala), as well as between bilateral medial/orbital frontal cortices and bilateral insula cortex.

Both of the above-discussed studies investigated patients at high and low risk for SUDEP, but no resting-state fMRI studies to date have included cases of actual SUDEP. Thus, a major limitation of both studies, is that the imaging correlates of risk factors associated with SUDEP are in fact reported, not necessarily the correlates of SUDEP itself. This issue remains a critical and inherent concern of all imaging studies into SUDEP, since cases of SUDEP are scarce, leading studies to rely on risk stratification of living subject datasets.

Despite their pitfalls, both experiments demonstrate altered networking among autonomic and breathing-related brain areas in those at high-risk for SUDEP. Larger studies, and investigations involving cases of SUDEP, may offer confirmation of disturbed connectivity and insights into the pathogenesis of SUDEP, which is still largely undefined. Overall, RS-fMRI has provided insights into connectivity changes in patients at high-risk of SUDEP which indicate altered communication among key brain regions contributing to autonomic and breathing regulatory processes. However, given the currently small body of literature, further work is required involving larger cohorts, healthy subjects, and victims of SUDEP to confirm initial work and characterize FC changes linked to SUDEP and other relevant clinical factors.

## Relationship Between Structural Changes and Functional Connectivity Disruptions

Some of the observed brain volume changes in SUDEP, and those at high risk for SUDEP, align with changes highlighted in the functional connectivity studies. For example, reduced volume within the thalamus observed in SUDEP and high-risk patients (20), as well as those with GTCS (22–24), appears to relate to the reduced connectivity of the thalamus (51, 52). Additionally, elevated volume and cortical thickening found in limbic structures such as the amygdala (20), bear resemblance to the increased FC of the bilateral mesial temporal structures in TLE patients at high risk (52).

Despite some homologous findings across structural and FC studies, further work is required for example to elucidate the link between volumetric changes and connectivity disruptions. In this respect, future studies should focus on combined volumetric and connectivity-based experiments [i.e., (22)] on the same cohorts of individuals and in larger datasets involving a diverse range of epilepsy sub-types.

## Further Considerations and Future Directions

### Relationships Between Regional Brain Volume and Clinical Epilepsy Variables

The volume of some brain structures has been found to correlate with clinical epilepsy-related variables, particularly in the thalamus. Disease duration, for example, correlates negatively with thalamic volume, as has been demonstrated extensively (22, 53–56), including gray matter within the pulvinar nuclei (20)—volume loss here is also associated with greater seizure frequency (57). Additionally, GTCS frequency correlates with cortical thickness of the cingulate and insula (32). However, both disease duration and seizure frequency are also major SUDEP risk factors (58); thus, a central objective for the field lies within disentangling the effects of the former from what is believed to be sequalae of the fatal event—representing a major challenge, since it is likely that they both contribute to the underlying mechanisms of SUDEP. This issue brings to light an overarching concern for all studies into SUDEP, namely the problem of defining imaging correlates of such under defined pathology. Modeling and controlling for clinical factors (e.g., disease duration, medications, and seizure frequency) in relation to regional brain volume changes are important aspects of SUDEP research, and should be carried out when considering brain alterations, since the observed volumetric changes may be related to presence of GTCS or epilepsy duration. Long-term prospective studies are needed to investigate all contributory factors of volume loss and connectivity alterations, including sex-specific alterations, as highlighted previously in a cortical thickness study of patients with GTCS (32).

### Future Studies

Given the relative rarity of SUDEP, multi-center collaborations, including such consortia as the Center for SUDEP Research (a center without walls initiative, funded by the National Institute of Neurological Disorders and Stroke), which bring together investigators from institutions across the US and UK and utilize open data sharing, will be crucial. Also, the integration of multi-modal imaging data, acquired prospectively seems essential for improved characterization of the relationship between structural and functional brain alterations: diffusion MRI, RS-fMRI, and T1- and T2-weighted MR images to investigate how volume changes and structural and functional connectivity alterations among regulatory structures arise and change in relation to clinical manifestations such as seizure frequency and disease duration. Additionally, the availability of ever larger retrospective datasets (including genetic data) for the wider research community would benefit efforts to better characterize SUDEP (including potential sub-types) and establish biomarkers using data-driven approaches.

Volumetric and morphological structural changes within the brainstem are a crucial aspect of research into SUDEP mechanisms, since the region contains many of the final common autonomic and respiratory pathways. Some anatomical properties of the brainstem and MR resolution limitations have restricted imaging studies to volumetric evaluation, either by gray and white matter segmentation, or amount of warping required to match a common template. Both techniques, which rely on T1-weighted contrast, may be insufficiently sensitive to detect underlying tissue changes within critical structures, particularly since many are small nuclei which lie on the border of white and gray matter. However, other newer procedures, such as quantitative MR T1/T2 ratio scans will enable assessment of myelin integrity, providing insights into allowing necessary evaluation of supportive tissue for neuronal processes in the brainstem and elsewhere.

### Combined Resting-State and Autonomic fMRI Studies

The observed disruptions of resting-state patterns in SUDEP patients mandate the assessment of failed vital functions, namely studies which incorporate concurrent recordings of autonomic and breathing patterns during fMRI scanning, enabling characterization of associations between resting FC and resting cardiovascular and breathing processes. In addition, conventional correlations of “evoked” fMRI changes to breathing and cardiovascular changes to triggered challenges, e.g., CO_2_ or hypoxia provocations, Valsalva maneuvers, cold pressor, or hand grip challenges may be useful to show magnitude of responses, timing delays or advancements between linked respiratory and cardiovascular areas. In other pathologic conditions, such as heart failure or congenital central hypoventilation syndrome, both distortions in timing and amplitude of linked structures appear (59, 60). Such “triggered” fMRI signal/physiological change correlations have the potential to show how dependencies between any given cortical or subcortical areas influence other areas; how time-delayed interactions can contribute to inappropriate timing of upper airway activation relative to diaphragmatic descent, leading to airway obstruction, or result in inappropriate or untimely compensatory blood pressure changes to challenges. Both scenarios can lead to physiologically-compromised circumstances, but the risk can be revealed by triggered fMRI studies.

### Relevance to the Identification of Preventative Interventions

The observed alterations in FC between brain structures which have the potential to elicit a cardiovascular or breathing crisis leading to SUDEP raise the issue of how 1 day we might be able to intervene in those dysfunctional pathways to avoid or overcome such crises. Potential targets for intervention are the neurotransmitters in the affected pathways or the enhancement of pathways for protective recovery circuitry. In addition, advances in neuromodulation procedures offer a means to intervene directly in disrupted functional pathways, which is of particular use here, since 31% of epilepsy patients are drug resistant (61). Neuromodulatory techniques, such as invasive stimulation of the vagus, has been effective for the decrease of seizure incidence [for a review, see (62)]; Furthermore non-invasive vagal stimulation can both reduce seizure incidence, and modify breathing and cardiovascular patterns (63–68). Therefore, the combination of identifying disrupted cardiovascular/respiratory functional pathways, and implementation of inputs from cranial nerves that will influence those pathways through non-invasive or invasive neuromodulatory techniques have the potential impact disrupted vital functions that lead to the fatal scenario in SUDEP.

## Summary and Conclusions

People who succumb to SUDEP, and those at risk, undergo regional brain structural changes and resting-state fMRI alterations between essential areas regulating cardiovascular and breathing control, indicating a structural and functional basis for impaired communication between areas necessary for recovery from compromised vital circumstances. The findings, although limited in sample sizes, are sufficiently apparent that indications of structural and functional changes may signal risk for SUDEP and shed light on underlying mechanisms. Moreover, both the structural and functional outcomes suggest means for potential interventions with specialized pharmacologic or neuromodulatory procedures. The proper characterization of the respective roles of the known risk factors, such as GTCS and disease duration, in relation to imaging findings can contribute to understanding SUDEP mechanisms, and warrant further investigation to disentangle clinical factors from what may be related to SUDEP.

## Author Contributions

LA and RH designed and prepared the manuscript. SL, LL, and BD contributed to preparation, critical review, and editing of the manuscript.

### Conflict of Interest Statement

The authors declare that the research was conducted in the absence of any commercial or financial relationships that could be construed as a potential conflict of interest. The handling editor declared a past co-authorship with one of the authors SL.
